# Preparation of Co_x_M_1−x_Al_2_O_4_/kaolinite (M = Zn, Ca, and Mg) composite pigments for ceramic glaze

**DOI:** 10.3389/fchem.2026.1879833

**Published:** 2026-09-01

**Authors:** Anjie Zhang, Mengmeng Wang, Xiangwei Xiao, Wenqiang Yan, Hao Yang, Bin Mu, Aiqin Wang

**Affiliations:** 1 College of Civil Engineering, Northwest Minzu University, Lanzhou, China; 2 Key Laboratory of Green Engineering Materials and Low-Carbon Construction, Lanzhou, Gansu, China; 3 Industrial Research Institute of Prefabricated Buildings and Energy-Saving Materials, Lanzhou, China; 4 School of Chemical Engineering, Zhengzhou University, Zhengzhou, China; 5 Key Laboratory of Clay Minerals of Gansu Province, Research Center of Resource Chemistry and Energy Materials, Lanzhou Institute of Chemical Physics, Chinese Academy of Sciences, Lanzhou, China

**Keywords:** ceramic glaze, cobalt aluminate, composite pigments, kaolinite, low cost

## Abstract

The high production cost of cobalt blue (CoAl_2_O_4_) pigments severely limits their wide application. Therefore, it is necessary to decrease the consumption of strategic cobalt resources to fabricate CoAl_2_O_4_ pigments with high blue intensity and good dispersion. Herein, Co_x_M_1−x_Al_2_O_4_/kaolinite (M = Zn, Ca, and Mg) composite pigments were prepared via coprecipitation followed by high-temperature crystallization using kaolinite and soluble metal salts as raw materials. It was revealed that the blue intensity of the composite pigments could be facilely adjusted by controlling the added amount and the type of the involved metal ions and the introduction of kaolinite. The incorporated metal ions could effectively change the cell parameters and further affect the electron transition of Co^2+^, while kaolinite provided a two-dimensional substrate to support the generated CoAl_2_O_4_ nanoparticles to improve the dispersion and reflective properties of visible light. Due to the synergistic effect of each component, the as-prepared composite pigments also exhibited satisfactory color properties and excellent thermal and suspension stability in ceramics. Therefore, this study was expected to develop a practical strategy for designing CoAl_2_O_4_ composite pigments with controllable blue intensity and low cobalt consumption for high-temperature ceramic glaze.

## Graphical Abstract

**Figure:**
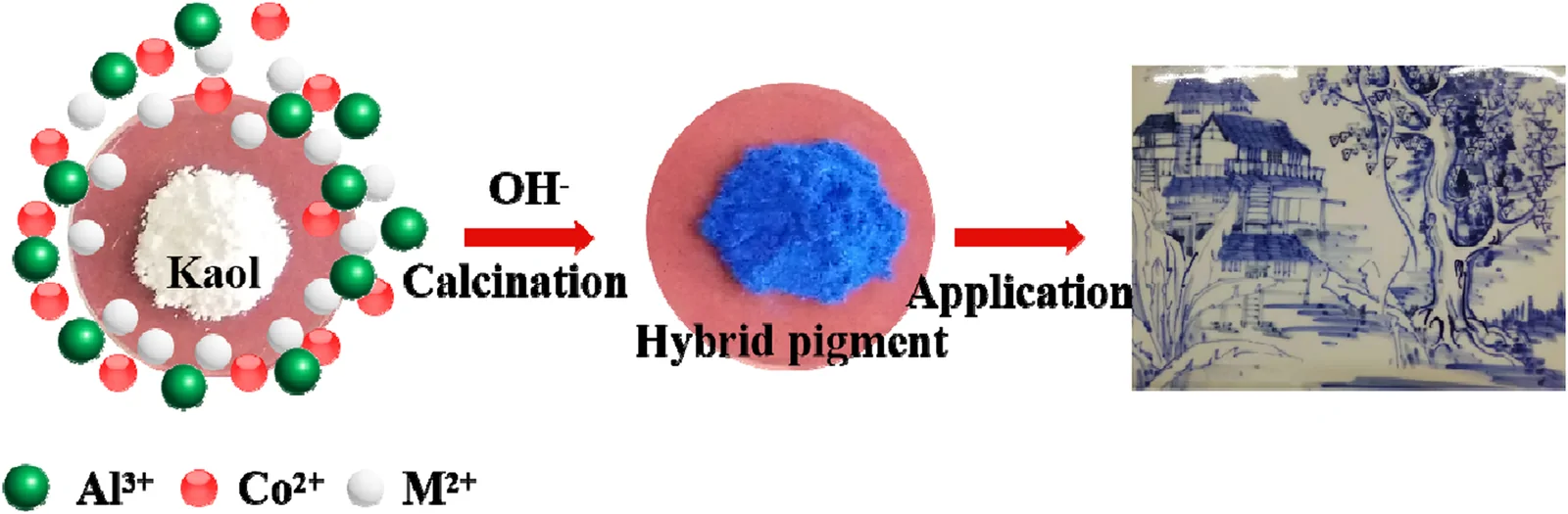

## Highlights

- A series of Co_x_M_1-x_Al_2_O_4_/kaolinine hybrid pigments were prepared for ceramic glaze.
- Hybrid pigments with lower cobalt consumption present excellent blue intensity.
- The color performance can be regulated by introducing kaolinite and metal ions.
- The formation and coloration mechanism of hybrid pigment were proposed.

## 1 Introduction

Cobalt aluminate (CoAl_2_O_4_), as a typical eco-friendly blue spinel-type inorganic pigment, has been widely used in ceramics, paints, and plastics due to its good chemical stability, high acid resistance, and excellent thermal stability (>1,200 °C) (Chapskaya et al., 2005; Chung and Masslth, 1980; Merino et al., 2015; He et al., 2018; Yoneda et al., 2018; Zhang et al., 2017; Zhang et al., 2018a; Álvarez-Docio et al., 2017). To meet different function and application requirements, different methods, such as solid-state, coprecipitation, and sol–gel, have been reported for the preparation of CoAl_2_O_4_ pigments. However, the high cost and serious particle agglomeration of CoAl_2_O_4_ pigments severely limited their scale applications (Llusar et al., 2001; Mimani and Ghosh, 2000; Mu et al., 2015; Zsoldos and Guczi, 1992).

Due to their abundance in nature, nontoxicity, low cost, and various morphologies, it has been confirmed that clay minerals can serve as ideal substrates for loading inorganic pigment nanoparticles (Wang et al., 2016; Todorova et al., 2014; Ezzatahmadi et al., 2017; Intachai et al., 2017). Therefore, a series of clay mineral-based CoAl_2_O_4_ composite pigments were prepared by incorporating kaolinite (Kaol), attapulgite, montmorillonite, halloysite, and sepiolite, adopting solid-phase, coprecipitation, microwave hydrothermal technology, and mechanochemical methods (Mu et al., 2015; Yang et al., 2025; Zhang et al., 2017; Zhang et al., 2018a; Zhang et al., 2018b; Zhang et al., 2018c; Zhang et al., 2018d; Sun et al., 2022; Zheng and Jian, 2015). It was revealed that the introduction of clay minerals decreased the particle size, improved the size distribution of CoAl_2_O_4_ nanoparticles, enhanced the color parameter, and reduced the production cost of CoAl_2_O_4_ pigments. However, the cost of CoAl_2_O_4_ pigments still needs to be continuously decreased to realize their wide applications (De Souza et al., 2009; Gholizadeh and Malekzadeh, 2017; Zhao et al., 2018), and it is imperative to explore a feasible process for the preparation of low-cost CoAl_2_O_4_ pigments with excellent color performance based on clay mineral-based CoAl_2_O_4_ composite pigments.

At present, the substitution of four-coordinated Co^2+^ by nontoxic metal ions (e.g., Mg^2+^, Zn, and Ni^2+^) is a feasible method to decrease the dosage of cobalt resources. Manikandan et al. (2016) synthesized blue Co_x_Zn_1−x_Al_2_O_4_ nanopigments after substituting Co^2+^ with Zn^2+^. Knyazev et al. (2017) prepared Co_x_Ni_1−x_Al_2_O_4_ nanopigments by the solid-state reaction, while Khattab et al. (2018) fabricated Co_x_Mg_1−x_Al_2_O_4_ pigments by the microwave combustion method. This suggests that doped metal ions with a similar ion radius to Co^2+^ can enter the tetrahedrally coordinated sites of CoAl_2_O_4_ to substitute Co^2+^, which contributes to decreasing the cost of cobalt blue and enhancing the color properties of cobalt blue pigments (Angeletti et al., 1977; Ahmed et al., 2008; Bosi et al., 2012; D’Ippolito et al., 2012; Dondi et al., 2014; Khattab et al., 2017). In the case of Ca^2+^, the coloring mechanism of the obtained pigments was different due to the large ion radius and lack of tetrahedral coordination, but it could effectively adjust the reflective properties of visible light (Yang et al., 2022). Therefore, the Co_x_M_1−x_Al_2_O_4_/Kaol (M = Zn, Ca, and Mg) composite pigments were synthesized via chemical coprecipitation and the subsequent calcination process integrating the superiority of cheap metal ions and clay minerals during the preparation of CoAl_2_O_4_ pigments. The effect of preparation conditions on the color parameters of composite pigments was also systematically investigated, including the solution pH, calcining temperature, calcining time, and the added amount and the type of different metal ions (Zn^2+^, Ca^2+^, Mg^2+^). Furthermore, the color of the composite pigments could be facilely adjusted by incorporating different metal elements. After the incorporation of Ca^2+^, the obtained composite pigments presented a blue color with a red hue, but Co_x_Zn_1−x_Al_2_O_4_ composite pigments were blue with a green hue. Based on the relevant characterizations, a possible coloring mechanism was proposed. Due to the similar ion radius of the tetrahedrally coordinated Co^2+^ and Mg^2+^ or Zn^2+^, Mg^2+^ or Zn^2+^ could enter the tetrahedrally coordinated sites of CoAl_2_O_4_ to substitute Co^2+^ and form the solid solutions of CoAl_2_O_4_–MgAl_2_O_4_ (or –ZnAl_2_O_4_). However, the ion radius of Ca^2+^ was higher than that of Co^2+^, and the introduction of Ca^2+^ led to the formation of homogeneous mixtures of CaAl_2_O_4_ and CoAl_2_O_4_. In addition, the application of the as-prepared composite pigments in ceramics was investigated.

## 2 Experiment and methods

### 2.1 Materials

Kaolinite was obtained from Longyan Kaolin Development Co., Ltd. (Fujian, China), which was first treated with 4 wt% HCl. The chemical compositions before and after treatment to remove associated minerals were analyzed by X-ray fluorescence and are provided in Supplementary Table S1. Co(NO_3_)_2_·6H_2_O, Ca(NO_3_)_2_·4H_2_O, Mg(NO_3_)_2_·6H_2_O, and Zn(NO_3_)_2_·6H_2_O were purchased from the Shanghai Reagent Factory (Shanghai, China), while Al(NO_3_)_3_·9H_2_O was obtained from Gelest. Sodium hydroxide and hydrochloric acid were provided by China National Medicines Co., Ltd.

### 2.2 Preparation of Co_x_M_1−x_Al_2_O_4_/Kaol composite pigments

First, the different amounts of Ca(NO_3_)_2_·4H_2_O were dissolved in 50 mL of deionized water under magnetic stirring at 400 rpm. Different amounts of Co(NO_3_)_2_·6H_2_O (the total added amounts of Ca(NO_3_)_2_·4H_2_O and Co(NO_3_)_2_·6H_2_O were 0.01 mol) and 0.02 mol of Al(NO_3_)_3_·9H_2_O were also added to an aqueous solution. Then, 1.09 g of Kaol (60 wt% of CoAl_2_O_4_) was added at 25 °C under magnetic stirring for 1 h to obtain a homogeneous solution. Second, a 3 M NaOH solution was added to adjust the pH value of the above mentioned solution to 10 under fast magnetic stirring for 2 h. The obtained solid was collected by centrifugation and washed in deionized water several times until the pH value was 7. Then, it was dried at 100 °C for 12 h. The obtained precursors were ground, sieved (400-mesh sieve), and calcined at different temperatures for 2 h. In order to optimize the conditions for the preparation of CoAl_2_O_4_ to obtain the best color parameters, the relevant conditions were systematically investigated, including the solution pH (6, 8, 10, and 12), calcining temperature (800 °C, 900 °C, 1,000 °C, 1,100 °C, and 1,200 °C), calcining time (0.5, 1, 1.5, and 2 h), the molar ratio of Co^2+^/M^2+^, and different metal ions (Ca^2+^, Mg^2+^, and Zn^2+^). The relevant preparation conditions are summarized in Table 1.

**TABLE 1:** Preparation conditions of the Co_x_M_1−x_Al_2_O_4_/Kaol composite pigment.

Factor	pH	Addition amount of Ca^2+^ (mol)	Temperature (°C)	Metal ions
pH	6	0.005	1,000	Ca^2+^
	8	0.005	1,000	Ca^2+^
	10	0.005	1,000	Ca^2+^
	12	0.005	1,000	Ca^2+^
Added amount of Ca^2+^ (mol)	10	0	1,000	Ca^2+^
	10	0.001	1,000	Ca^2+^
	10	0.002	1,000	Ca^2+^
	10	0.003	1,000	Ca^2+^
	10	0.004	1,000	Ca^2+^
	10	0.005	1,000	Ca^2+^
	10	0.006	1,000	Ca^2+^
	10	0.007	1,000	Ca^2+^
	10	0.008	1,000	Ca^2+^
	10	0.009	1,000	Ca^2+^
	10	0.01	1,000	Ca^2+^
Calcining temperature (°C)	10	0.005	800	Ca^2+^
	10	0.005	900	Ca^2+^
	10	0.005	1,000	Ca^2+^
	10	0.005	1,100	Ca^2+^
	10	0.005	1,200	Ca^2+^
Calcining time (h)	0.5	0.005	1,100	Ca^2+^
	1	0.005	1,100	Ca^2+^
	1.5	0.005	1,100	Ca^2+^
	2	0.005	1,100	Ca^2+^
Metal ions	10	0.005	1,000	Ca^2+^
	10	0.005	1,000	Mg^2+^
	10	0.005	1,000	Zn^2+^

### 2.3 Characterization

Transmission electron microscopy (TEM, JEM-1200EX/S, JEOL) was used to observe the morphologies of samples. The XRD patterns of the samples were obtained on an X’pert PRO diffractometer (*λ* = 1.54060 Å) with a step interval of 0.167° between 10° and 80° (*2θ*) at a scanning rate of 0.05°/s^−1^ at room temperature. The Commission Internationale de l’Eclairage (CIE) of the samples were measured on X-Rite, Ci 7800, using the *CIE*-1976 *L* ^*^, *a* ^*^, *b* ^*^ colorimetric method, in which *L* ^*^ is the lightness axis (from 0 black to 100 white); *a* ^*^ is the red–green axis (negative values indicate greenness and positive values indicate redness); and *b* ^*^ is the yellow–blue axis (negative values indicate blueness and positive values indicate yellowness).

### 2.4 Preparation of ceramic glaze

A fusion mass was prepared using lead oxide, quartz, borax, and other components; the as-prepared Co_0.5_Ca_0.5_Al_2_O_4_/Kaol composite pigments were added to it, and the mixture was ground in a ball mill for 12 h to produce the pigment paste (traditionally referred to as “cai gu”). Next, the frankincense oil or adhesive was then mixed in as a binder. A white or monochrome glazed porcelain body that had already undergone high-temperature firing (approximately 1,200 °C–1,300 °C) was selected. The ceramic surface was carefully wiped with alcohol or warm water to remove oil stains and dust, ensuring that the pigment adhered tightly. Painting was subsequently applied to the ceramic body, which was then carefully placed into an electric kiln and heated at a rate of 2.5 °C/min to 800 °C, maintained for 15–30 min, and finally removed after natural cooling.

## 3 Results and discussion

### 3.1 Characterization of Kaol-HP

**FIGURE 1:**
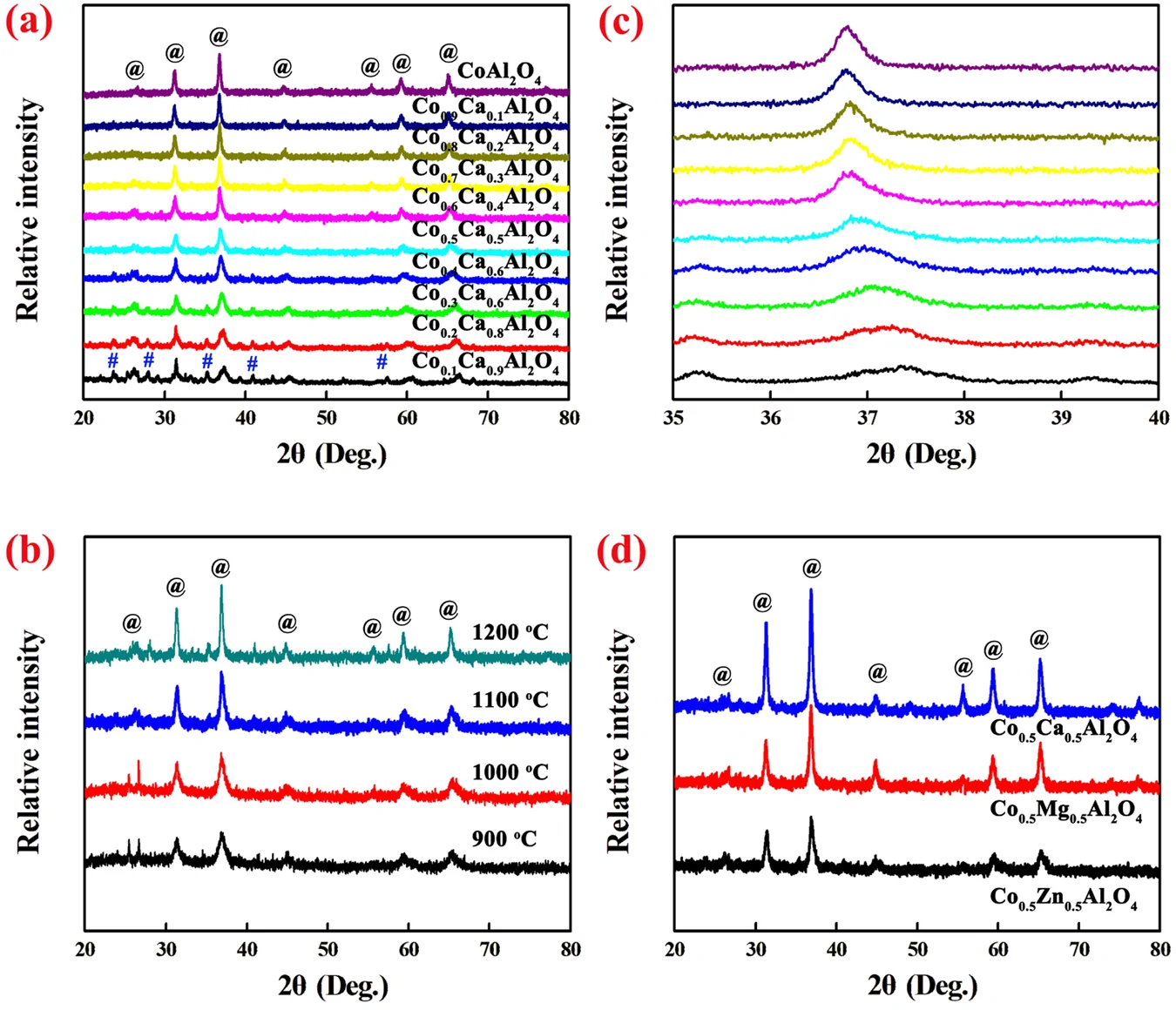
XRD patterns of Co_0.5_Ca_0.5_Al_2_O_4_/Kaol at different preparation conditions: **(a)** different molar ratios of Co/Al calcinated at 1,100 °C, **(b)** partially enlarged view of XRD patterns of Figure 2a, **(c)** different calcining temperatures, and **(d)** different doped elements calcinated at 1,100 °C.

Figure 1 shows the XRD patterns of Co_x_Ca_1−x_Al_2_O_4_/Kaol under different preparation conditions. Figure 1a shows that the peaks located at *2θ* = 31.1°, 36.8°, 44.8°, 49.0°, 55.5°, 59.2°, and 65.2° correspond to the (220), (311), (400), (331), (422), (511), and (440) crystal planes of spinel CoAl_2_O_4_ (JCPDs card No. 10-0458), respectively (Abaide et al., 2015; Zhang et al., 2018c). Furthermore, the observed peaks located at *2θ* = 23.7°, 27.9°, 35.3°, 40.8°, and 57.6° were assigned to the typical diffraction peaks of CaAl_2_O_4_ (JCPDs card No. 23-1036). The observed peaks located at *2θ* = 26.3°, 35.49°, and 60.7° were also assigned to the typical diffraction peaks of mullite (JCPDs card No. 79-1453) (see Supplementary Figure S1). As the Ca^2+^ dosage was lower than 0.004 mol, the main crystal phases in Co_x_Ca_1−x_Al_2_O_4_/Kaol were CaAl_2_O_4_ and CoAl_2_O_4_. As the Ca^2+^ dosage was 0.005 mol, the typical diffraction peaks of CaAl_2_O_4_ vanished. Moreover, when Kaol was turned into mullite at 1,100 °C, the relative intensity of the characteristic reflection peaks of CoAl_2_O_4_ increased with the increase in the added amount of Ca^2+^, and the FWHM broadened accompanied with a slight shift to a low angle.

The crystallite sizes and cell parameters of CoAl_2_O_4_ are presented in Table 2. The crystallite size is calculated using the Sherrer equation (Equation 1), while the cell parameter is calculated based on the *hkl (311)* crystal plane according to the following formula for the cubic lattice (Equation 2):

$$D=\frac{0.89\times \lambda }{B\times \cos\theta }$$ (1)

where *D* is the crystallite size (nm), *λ* is the wavelength (Cu Ka), and *B* is the corrected half-width obtained using quartz as a reference. *B* = *B*_*m*_ − *B*_*s*_, where *B*_*m*_ refers to the test results and *B*_*s*_ is obtained by testing a reference substance. *θ* is the diffraction angle of the most intense diffraction peak (Ragupathi et al., 2015):

$$a=\frac{\lambda \;}{2\cos\;\theta \;}\times \sqrt{h^{2}+k^{2}+l^{2},}$$ (2)

where *a* is the cell parameter, *λ* is the wavelength (Cu Ka), and *θ* is the diffraction angle of the most intense diffraction peak (Koroleva, 2004).

**TABLE 2:** Color parameters, crystallite sizes, and cell parameters of the Co_x_Ca_1−x_Al_2_O_4_/Kaol composite pigment.

Sample	Full Width at Half Maximum (FWHM) (rad)	Crystallite size (nm)	Cell parameters (Ǻ)
Co_0.1_Ca_0.9_Al_2_O_4_	0.612	13	8.116 ± 0.002
Co_0.2_Ca_0.8_Al_2_O_4_	0.603	14	8.113 ± 0.001
Co_0.3_Ca_0.7_Al_2_O_4_	0.562	15	8.112 ± 0.001
Co_0.4_Ca_0.6_Al_2_O_4_	0.531	16	8.110 ± 0.002
Co_0.5_Ca_0.5_Al_2_O_4_	0.451	19	8.109 ± 0.002
Co_0.6_Ca_0.4_Al_2_O_4_	0.35	25	8.106 ± 0.001
Co_0.7_Ca_0.3_Al_2_O_4_	0.353	24	8.106 ± 0.002
Co_0.8_Ca_0.2_Al_2_O_4_	0.316	27	8.105 ± 0.001
Co_0.9_Ca_0.1_Al_2_O_4_	0.288	30	8.104 ± 0.001
CoAl_2_O_4_	0.273	32	8.104 ± 0.002

It is clearly observed that the crystallite sizes of Co_x_Ca_1−x_Al_2_O_4_ increase with the increase in the added amount of Ca^2+^, while the cell parameter of CoAl_2_O_4_ changes from 8.116 to 8.104 Ǻ, which results from the fact that doping ions with larger ionic radii increase the cell parameter, whereas doping ions with smaller ionic radii decrease the cell parameter. As shown in Table 2, the cell parameters of the as-prepared Co_x_Ca_1−x_Al_2_O_4_/Kaol composite pigments prepared by Ca doping are larger than those of CoAl_2_O_4_ pigments, which might be ascribed to the ionic radius of the doped Ca^2+^ being larger than that of Co^2+^. The increase in the relative intensity of typical diffraction is due to the increase in the crystallinity degree of CoAl_2_O_4_.

Figure 1c shows the XRD patterns of the Co_0.5_Ca_0.5_Al_2_O_4_/Kaol composite pigment calcined at different temperatures. With the increase in the calcining temperature from 900 °C to 1,200 °C, the relative intensity of the diffraction peaks gradually increased as the peak width became narrow, indicating an increase in the crystallinity degree of Co_0.5_Ca_0.5_Al_2_O_4_. In addition, the relative intensity of the diffraction peak of quartz decreased when the calcining temperature was up to 1,000 °C. With the continued increase in the calcining temperature, the peaks of quartz almost disappeared, which might be ascribed to the transformation to an amorphous form (Ran et al., 2016). The effect of the doping elements on the formation process of spinel CoAl_2_O_4_ is depicted in Figure 1d. It could be found that the relative intensity of the diffraction peaks of Co_0.5_Ca_0.5_Al_2_O_4_ is the strongest, indicating the enhancement in the crystallinity degree of CoAl_2_O_4_ due to the doping of Ca^2+^.

**FIGURE 2:**
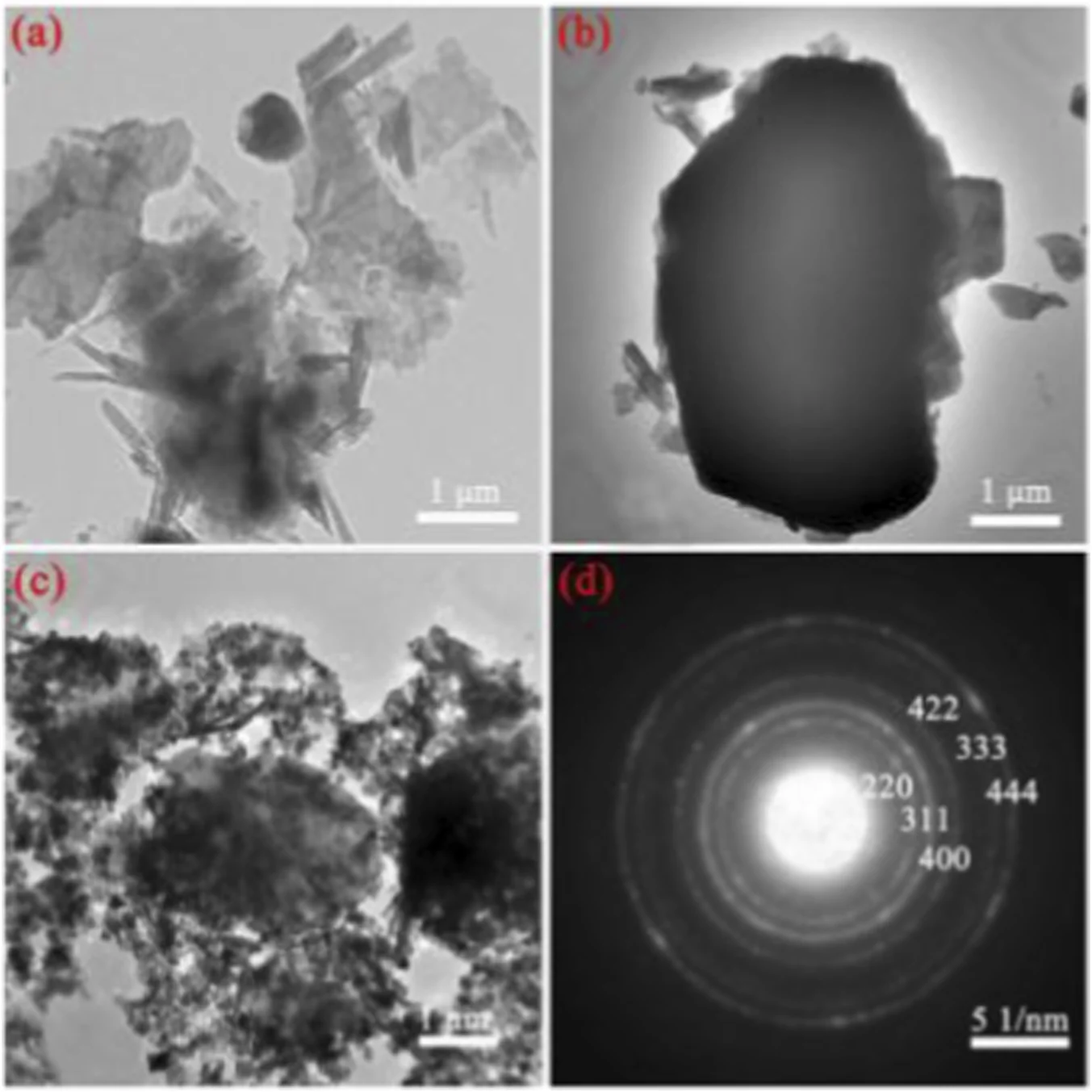
TEM of **(a)** Kaol, **(b)** Kaol, and **(c)** Co_0.5_Ca_0.5_Al_2_O_4_/Kaol calcined at 1,100 °C and **(d)** the selected area electron diffraction pattern of Co_0.5_Ca_0.5_Al_2_O_4_/Kaol.

**FIGURE 3:**
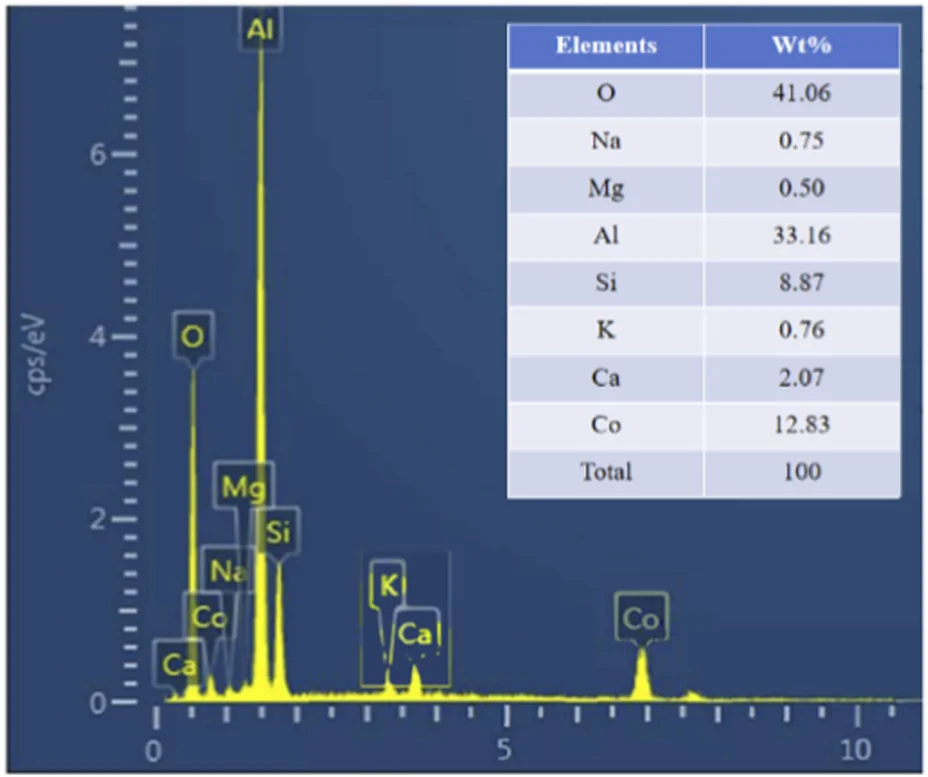
EDX of the Co_0.5_Ca_0.5_Al_2_O_4_/Kaol composite pigment.

**FIGURE 4:**
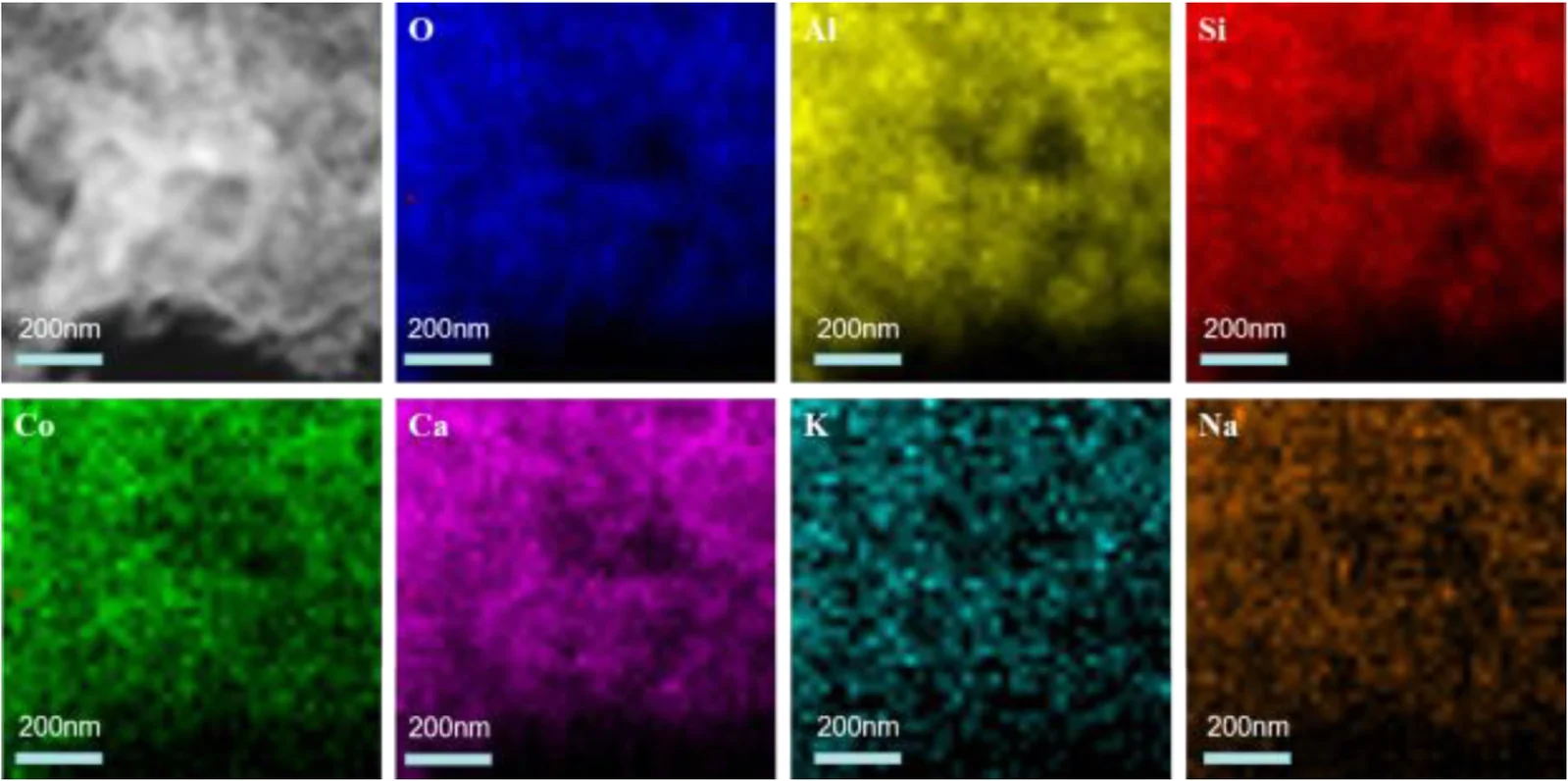
Mapping of the Co_0.5_Ca_0.5_Al_2_O_4_/Kaol composite pigment.

As illustrated in Figure 2a, Kaol presents a typical lamellar tubular Hal, and the length of Hal is approximately 0.2–2.0 μm, while the external and inner diameters are approximately 50–80 and 20–50 nm, respectively (Zhang et al., 2018a; Zhang et al., 2019). After being calcined at 1,100 °C, the lamellar morphology of Kaol (Figure 2b) and the Kaol in the composite pigment (Figure 2c) remained, and the hollow structure of hal disappears accompanied with the decrease in the length and the increase in the diameter, which might be related to the phase transformation of Hal under high temperatures. Interestingly, the electron diffraction pattern of the selected area of Kaol-HP calcined at 1,100 °C confirms the formation of Co_0.5_Ca_0.5_Al_2_O_4_ nanoparticles (Figure 2d) (Ouahdi et al., 2005; Mindru et al., 2010); thus, the observed phenomenon that the surface of lamellar morphology becomes coarse is due to the Co_0.5_Ca_0.5_Al_2_O_4_ nanoparticles (with a diameter of approximately 10–20 nm) uniformly anchored on the surface of Kaol (Figure 2c). In addition, it was found that the Co_0.5_Ca_0.5_Al_2_O_4_/Kaol composite pigment was mainly composed of Co, Al, O, Si, and Ca elements according to the EDX spectra of the Co_0.5_Ca_0.5_Al_2_O_4_/Kaol composite pigment calcined at 1,100 °C (Figure 3). All elements presented a uniform distribution with a well-defined structure as observed from the elemental mappings of the Co_0.5_Ca_0.5_Al_2_O_4_/Kaol composite pigment (Figure 4). It was worth noting that the uniform distribution of the Co element was an indication of the uniform loading of Co_0.5_Ca_0.5_Al_2_O_4_ nanoparticles on the surface of Kaol. Moreover, the EDX analysis of the Co_0.5_M_0.5_Al_2_O_4_/Kaol composite pigment detected only 2.07 wt% Ca, compared with 12.83 wt% Co. This result does not satisfy the Co/Ca ratio of 1:1. Since the EDX analysis of heterogeneous ceramic powders is semiquantitative and depends on surface and local detection conditions, it cannot verify the bulk stoichiometry of the material. X-ray fluorescence (XRF) analysis data for the Co_0.5_M_0.5_Al_2_O_4_/Kaol composite pigment are presented in Supplementary Table S2 (see ESI). The XRF results for the Co_0.5_M_0.5_Al_2_O_4_/Kaol composite pigment indicate that the Co/Ca ratio is close to 1:1, which further confirms the formation of the Co_0.5_M_0.5_Al_2_O_4_/Kaol composite pigment. Moreover, Supplementary Table S2 shows that the Co wt% of the Co_0.5_M_0.5_Al_2_O_4_/Kaol composite pigment was 11.87, which is far lower than that of CoAl_2_O_4_/Kaol and CoAl_2_O_4_. A lower cobalt content corresponds to a lower production cost. Therefore, the introduction of low-cost metal ions and clay minerals during the preparation of CoAl_2_O_4_ pigments reduces the production cost of these pigments.

**FIGURE 5:**
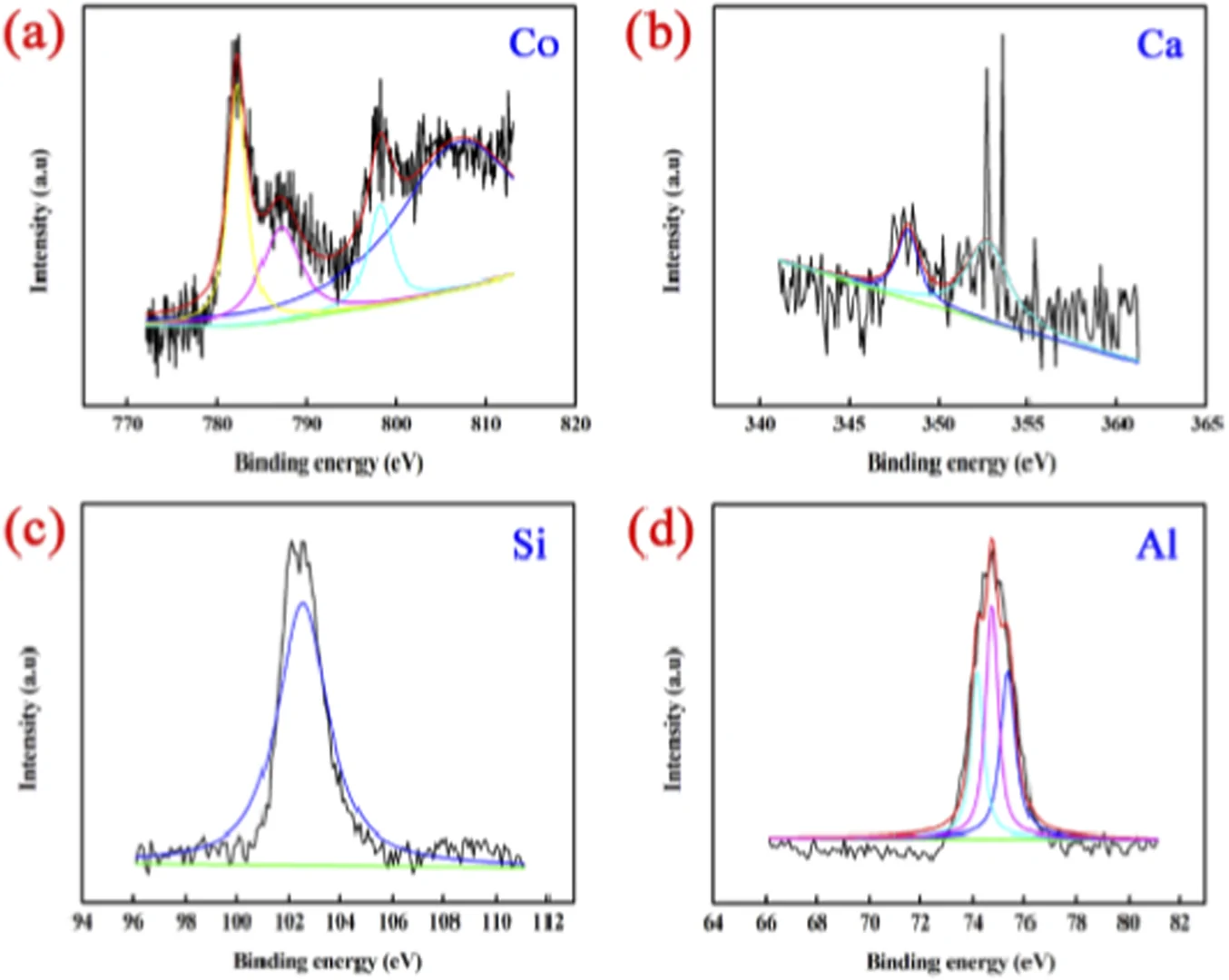
XPS spectra of the Co_0.5_Ca_0.5_Al_2_O_4_/Kaol composite pigment: **(a)** Co 2p, **(b)** Ca 2p, **(c)** Si 2p, and **(d)** Al 2p.

The XPS spectra of the Co_0.5_Ca_0.5_Al_2_O_4_/Kaol composite pigment calcined at 1,100 °C were employed to comparatively analyze the chemical state of the elements in the near-surface region (Figure 5) and the effects of the incorporation of Ca^2+^ on the crystal structure and color performance of the CoAl_2_O_4_ pigments. The C 1s binding energy (BE) peak at 284.8 eV was used to correct the obtained fine spectrum. The locations of BE peaks of Co 2p and Al 2p and the ratio of the integral area of the fitted Co 2p BE peaks are listed in Table 3. From the XPS spectra of Co 2p of the Co_0.5_Ca_0.5_Al_2_O_4_/Kaol composite pigment (Figure 5a), it was clearly observed that the peaks of Co 2p were fitted into four different types of peaks. p2/p1 and p4/p3 were the ratios of the integral areas of peaks 2 and 1 and peaks 4 and 3, respectively. Peaks 1 and 3 were assigned to Co 2p_3/2_ and Co 2p_1/2_, respectively; peaks 2 and 4 correspond to the shake-up satellites of Co 2p_3/2_ and Co 2p_1/2_, respectively (Liu et al., 2018; Sale, 2015; Petitto et al., 2008). The presented BE peaks of Co 2p_3/2_ at 782.3 eV and Co 2p_1/2_ at 798.1 eV with an energy difference of 15.8 eV confirmed the presence of Co^2+^. The two peaks observed at 787.1 and 807.1 eV are the satellite peaks for Co 2p_3/2_ and Co 2p_1/2_, respectively. In general, Co ions possess two different valence states, namely, the high-spin Co^2+^ and low-spin Co^3+^. The more intense satellites were typical for high-spin Co^2+^, which was attributed to electron correlations and final state effects. Generally, the greater the intensity of satellites, the more Co^2+^ occupied the tetrahedral sites in the spinel CoAl_2_O_4_ (Cai et al., 2002; Morris and Martin, 1970; Peymannia et al., 2015; Duan et al., 2011); thus, p2/p1 and p4/p3 represented the content of Co^2+^ occupying the tetrahedral sites in the overall Co element. It can be observed that the values of p2/p1 and p4/p3 increased after the incorporation of Ca^2+^ compared with the CoAl_2_O_4_/Kaol pigment and the CoAl_2_O_4_ calcined at 1,100 °C, which suggested the higher content of Co^2+^ in tetrahedral sites, and it is the reason why the prepared composite pigments exhibited a brighter blue color (Zsoldos and Guczi, 1992). It is well known that Co^2+^ and Al^3+^ occupied the tetrahedral and octahedron sites, respectively, in the spinel structure of CoAl_2_O_4_ (Chung et al., 1980). However, Co^2+^ might partially enter the octahedral sites at high temperatures, and Al^3+^ can also occupy the tetrahedral sites to form an inverse spinel structure; thus, the binding energy location of Co 2p shifts toward the low binding energy (Tang et al., 2018).

**TABLE 3:** Binding energy (eV) of Co 2p and Al 2p of the Co_0.5_Ca_0.5_Al_2_O_4_/Kaol composite pigment, CoAl_2_O_4_/Kaol-1100, and CoAl_2_O_4_-1100.

Sample	BE of Co 2p (eV)				Ratio of the area		BE of Al 2p (eV)
	Peak 1	Peak 2	Peak 3	Peak 4	p2/p1	p4/p3	
Co_0.5_Ca_0.5_Al_2_O_4_/Kaol	782.3	787.1	798.1	807.1	0.84	7.36	75.4
							74.7
							74.1
CoAl_2_O_4/_Kaol-1100	781.8	786.2	797.2	805.0	0.71	6.69	75.5
							74.8
							74.1
CoAl_2_O_4_-1100	781.9	786.4	797.3	803.2	0.31	0.96	74.2
							75.0

As shown in Figure 5d and Table 3, the Al 2p of the Co_0.5_Ca_0.5_Al_2_O_4_/Kaol composite pigment could be divided into three BE peaks. According to Zhang et al. (2010), the Al 2p BE peak of Kaol is located at 75.5 eV, while the Al 2p peaks of the Co_0.5_Ca_0.5_Al_2_O_4_/Kaol composite pigment at 74.1 and 74.7 eV were ascribed to the tetrahedral and octahedral sites of the spinel CoAl_2_O_4_, respectively. Compared with CoAl_2_O_4_-1100 and CoAl_2_O_4/_Kaol, the peak location of Al 2p of Kaol-HP also shifted toward the low binding energy, confirming that the as-prepared Co_0.5_Ca_0.5_Al_2_O_4_/Kaol composite pigment partially exhibited an inverted spinel structure (Tang et al., 2017). In the case of the Ca 2p of Co_0.5_Ca_0.5_Al_2_O_4_/Kaol composite pigment, the two divided BE peaks at 348.3 and 352.7 eV corresponded to Kaol and Co_0.5_Ca_0.5_Al_2_O_4_, respectively, which indicated that Ca^2+^ successfully participated in the formation of the composite pigment.

### 3.2 Effect of the preparation conditions

**FIGURE 6:**
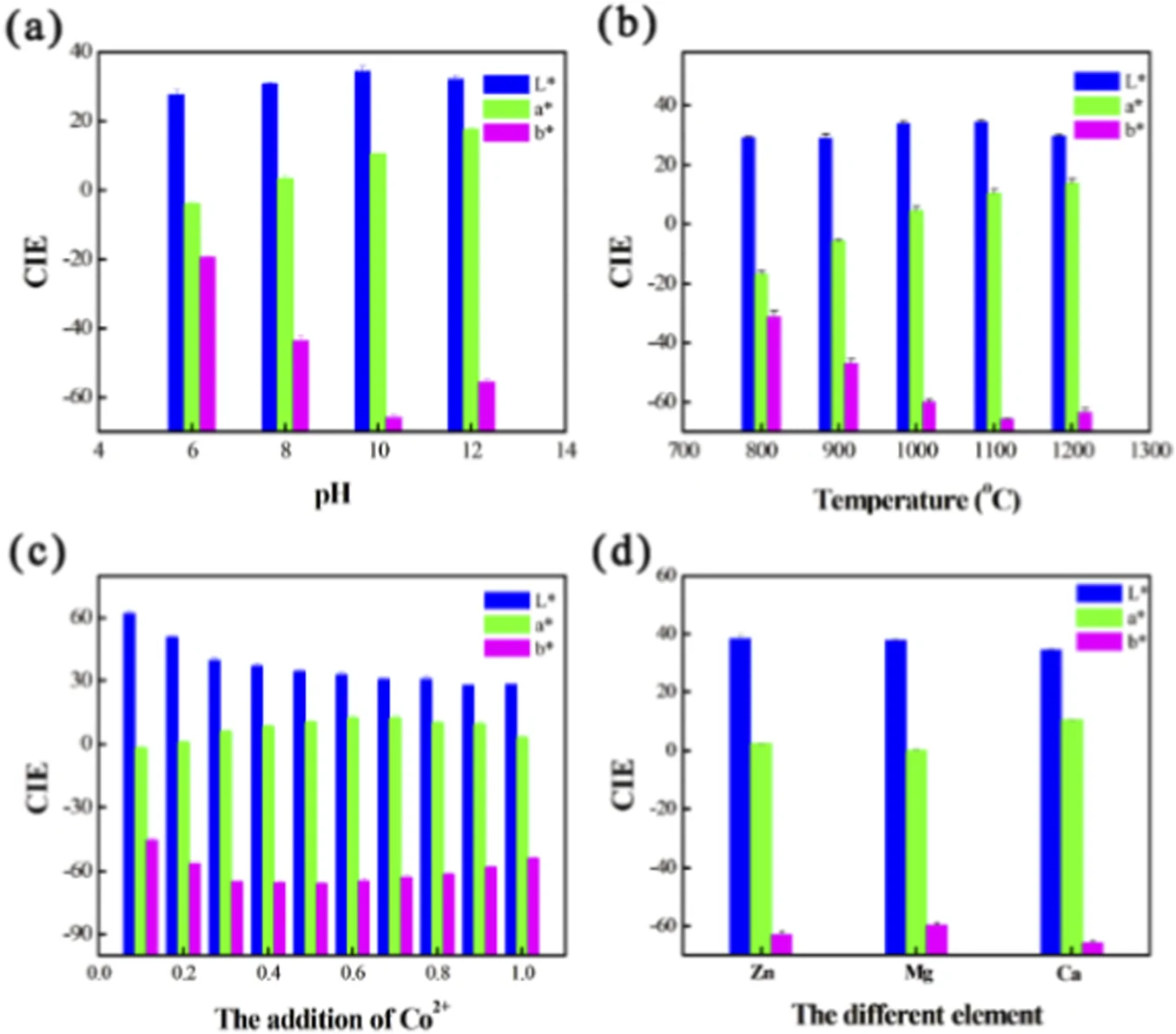
*CIE* of Co_0.5_Ca_0.5_Al_2_O_4_/Kaol at different **(a)** pH, **(b)** calcining temperatures, **(c)** ratios of Co/Al, and **(d)** doped elements.

Figure 6a shows the relationship between the *CIE* and the solution pH value. It indicates that the Co_0.5_Ca_0.5_Al_2_O_4_/Kaol composite pigment prepared at pH = 10 exhibited optimum color properties (*L* ^*^ = 34.32, *a* ^*^ = 10.39, and *b* ^*^= −65.84). Generally, Co^2+^ in the reaction system does not precipitate completely when the pH is below 10. However, the higher the pH value, the greater the Al loss due to the dissolution of Al(OH)_3_ in alkaline conditions (Zhang et al., 2018c; Zhang et al., 2019). Therefore, the pH value of the solution is buffered close to 10 for the preparation of composite pigments.

Figure 6b shows the relationship between the *CIE* and the calcining temperature of the Co_0.5_Ca_0.5_Al_2_O_4_/Kaol composite pigment. It could be found that the *L* ^*^ values of the Co_0.5_Ca_0.5_Al_2_O_4_/Kaol composite pigment increased with the increase in the calcining temperature. When the calcining temperature increases to 1,200 °C, the *L* ^*^ value of Kaol-HP begins to decrease. Interestingly, the *b* ^*^ values first decreased and then increased, indicating that the prepared Co_0.5_Ca_0.5_Al_2_O_4_/Kaol composite pigment exhibited optimal color performance at a calcining temperature of 1,100 °C, which might be due to the change in the microstructure of the composite pigments at higher temperatures (Yang et al., 2023; Zhang et al., 2018c).

Supplementary Figure S2 (see ESI) shows the reflectance spectra of the as-prepared Co_0.5_Ca_0.5_Al_2_O_4_/Kaol composite pigment at different temperatures. All samples present the reflection bands at 440–500 nm (blue to blue–green) and absorption bands at 550–650 nm (green to red) (He and Becker, 1997; He et al., 2018; Torkian and Daghighi, 2014), except for the Co_0.5_Ca_0.5_Al_2_O_4_/Kaol composite pigment calcined at 900 °C. The characteristic reflection peak at approximately 480 nm is ascribed to the characteristic curve of CoAl_2_O_4_. The absorption curves exhibit three absorption peaks at approximately 540, 590, and 630 nm in the range of 500–650 nm, which are attributed to tetrahedral coordinated Co^2+^ (Kurajica et al., 2012; Gomesan et al., 2015; Tahereh et al., 2016). In addition, the intensity of the reflection peaks increased with the increase in the calcining temperature until 1,100 °C, but it decreased as the calcining temperature reached 1,200 °C, which is consistent with the change in their color parameters (Figure 6b).

As shown in Figure 6c, it was clearly observed that the *L* ^*^ values of the Co_x_Ca_1−x_Al_2_O_4_/Kaol composite pigment decreased with the increase in the addition of Co^2+^. However, the *b* ^*^ values first decreased and then increased, and the minimum *b* ^*^ values reached −65.84 when the addition of Co was 0.005 mol. Therefore, the hybrid pigments prepared at 1,100 °C exhibited excellent color properties (*L* ^*^ = 34.32, *a* ^*^ = 10.39, and *b* ^*^ = −65.84), which were higher than those of the reported works and commercial cobalt blue pigments (Supplementary Table S3; see ESI). The influence of different calcining times on the *CIE* of the Co_0.5_Ca_0.5_Al_2_O_4_/Kaol composite pigment is shown in Supplementary Figure S3 (see ESI). Supplementary Figure S3a shows that the *L* ^*^ values of the Co_0.5_Ca_0.5_Al_2_O_4_/Kaol composite pigment increased with the increases in calcining time and that the *b* ^*^ values of the Co_0.5_Ca_0.5_Al_2_O_4_/Kaol composite pigment decreased with the increases in calcining time. This is primarily due to the increased crystallinity of the Co_0.5_Ca_0.5_Al_2_O_4_ pigment as the calcining time increases (Supplementary Figure S3b).

**FIGURE 7:**
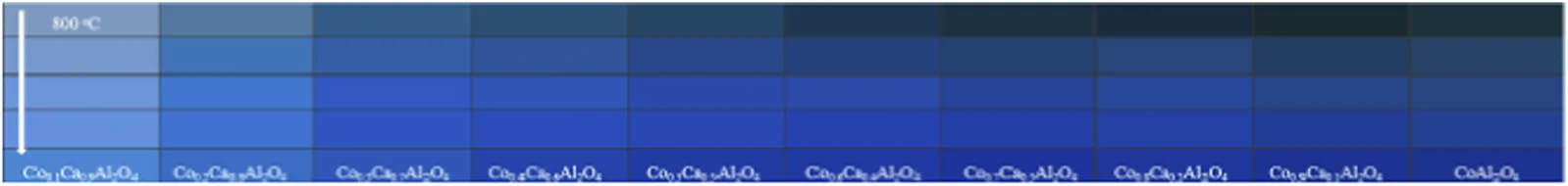
Digital photos of Co_1−x_Ca_x_Al_2_O_4_ at different calcining temperatures.

**FIGURE 8:**
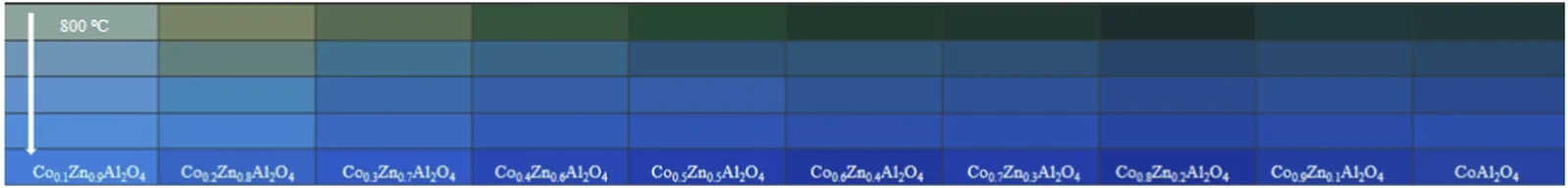
Digital photos of Co_1−x_Zn_x_Al_2_O_4_ at different calcining temperatures.

**FIGURE 9:**
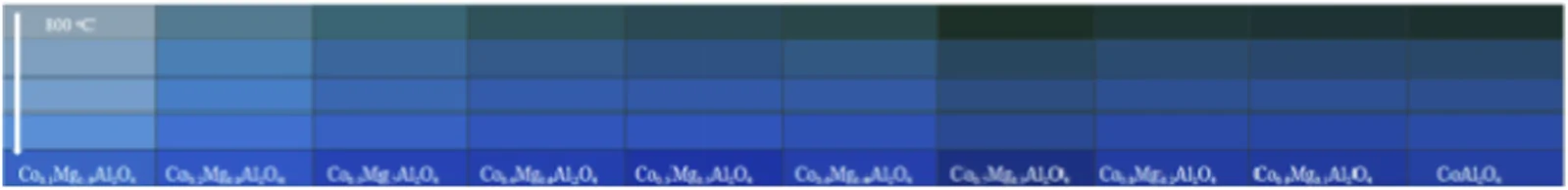
Digital photos of Co_1−x_Mg_x_Al_2_O_4_ at different calcining temperatures.

Figure 6d shows the influence of different metal ions on the *CIE* of the composite pigments. The *CIE* of Co_x_Ca_1−x_Al_2_O_4_/Kaol composite pigments prepared under different conditions are given in Supplementary Figures S4-S6 (see ESI), and the digital photos of the pigments prepared under different conditions are shown in Figures 7–9. It could be found that the composite pigments after the incorporation of different metal ions appeared nearly uniform blue according to the *L* ^*^ and *b* ^*^ values. In addition, the introduction of different metal ions obviously improved the *L* ^*^ and *b* ^*^ values compared with those of CoAl_2_O_4_/Kaol composite pigments prepared under the same conditions. The *a* ^*^ values of the composite pigments prepared with Mg^2+^ are in good agreement with those of CoAl_2_O_4_/Kaol composite pigments. It is worth noting that the *a* ^*^ values of the composite pigments prepared with Ca^2+^ are higher than those of the CoAl_2_O_4_/Kaol composite pigment, while the *a* ^*^ values of the composite pigments prepared with Zn^2+^ are lower than those of the CoAl_2_O_4_/Kaol composite pigments prepared under the same control conditions. Therefore, it can be concluded that the introduction of different doped elements can well control the hue of the pigments. The changes in color parameters can be attributed to the same ionic radius between the tetrahedrally coordinated Co^2+^ and the doped Mg^2+^ and Zn^2+^ ions. Mg^2+^ and Zn^2+^ can enter the tetrahedrally coordinated sites of CoAl_2_O_4_ to substitute Co^2+^ (Ahmed et al., 2008; Bosi et al., 2012; Dondi et al., 2014; D’Ippolito et al., 2012; Khattab et al., 2017). However, the cubic spinel CoAl_2_O_4_ and CaAl_2_O_4_ could not form a solid solution with CoAl_2_O_4_ by replacing Co^2+^ with Ca^2+^ owing to the remarkable differences in the ionic radius and coordination number between Ca^2+^ and Co^2+^; thus, the blue intensity regulation of the composite pigment after the incorporation of Ca^2+^ may be related to the interaction between the CoAl_2_O_4_ and CaAl_2_O_4_ crystals during the crystallization process (Yang et al., 2022). Furthermore, the color of the composite pigments could be facilely adjusted by incorporating different metal elements. After the incorporation of Ca^2+^, the obtained composite pigments presented a blue color with a red hue, but Co_x_Zn_1−x_Al_2_O_4_ composite pigments were blue with a green hue.

### 3.3 Application of the Co_0.5_Ca_0.5_Al_2_O_4_/Kaol composite pigments in ceramics

**FIGURE 10:**
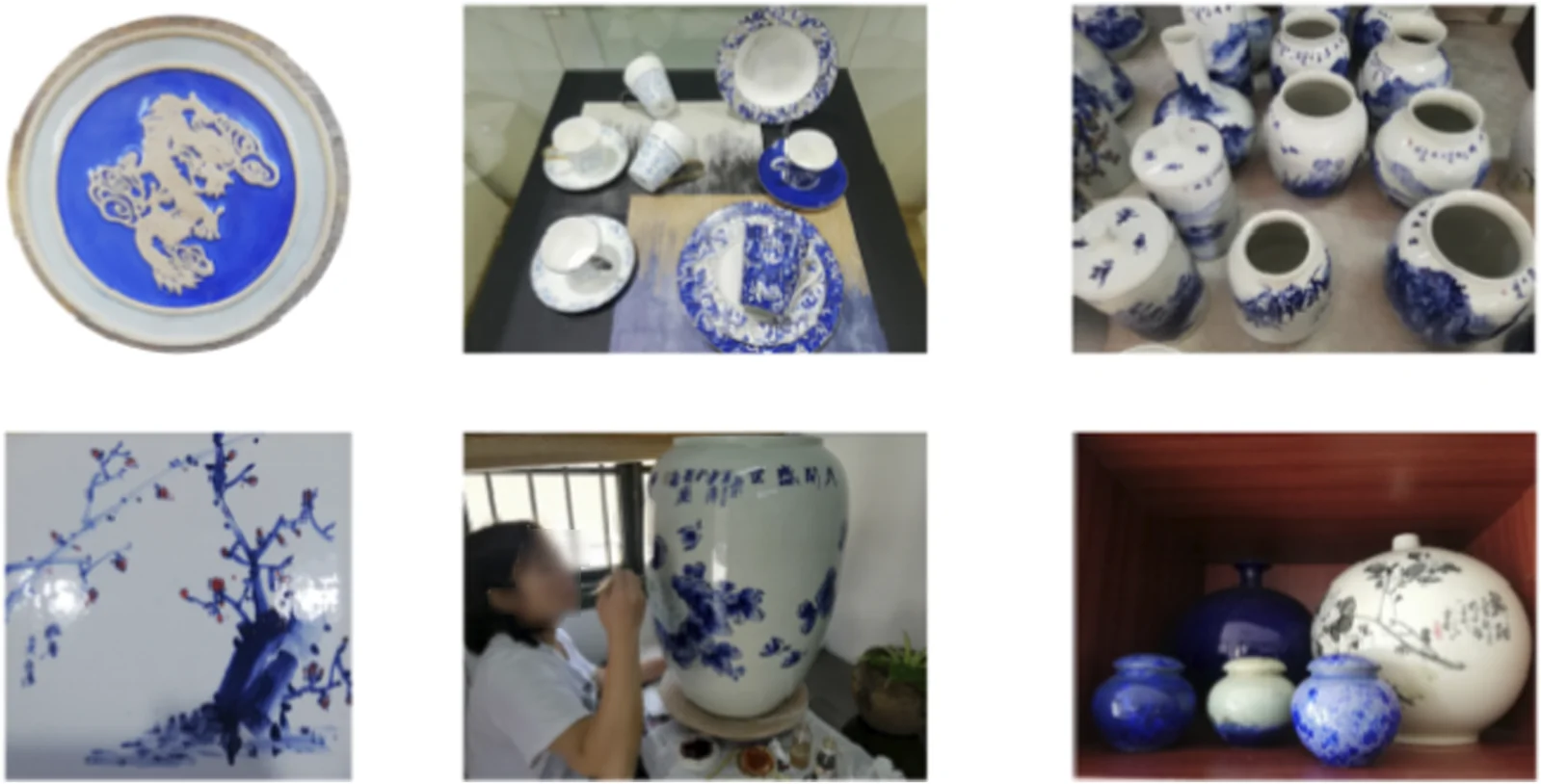
Application of the Co_0.5_Ca_0.5_Al_2_O_4_/Kaol composite pigment in ceramics.

Supplementary Table S7 (see ESI) shows the performance parameters of the Co_0.5_Ca_0.5_Al_2_O_4_/Kaol composite pigment. The results revealed that the Co_0.5_Ca_0.5_Al_2_O_4_/Kaol composite pigments exhibited excellent resistance. CoAl_2_O_4_ pigments are widely employed as a colorant in ceramics at high temperatures, so the as-prepared Co_0.5_Ca_0.5_Al_2_O_4_/Kaol composite pigments was applied to color the ceramics to investigate their heating resistance. As shown in Figure 10, it is clearly observed that the Co_0.5_Ca_0.5_Al_2_O_4_/Kaol composite pigment combines well with the ceramic and has a benign dispersion on the ceramic surface. In addition, the surface is smooth without any fractures. Therefore, the low-cost Co_0.5_Ca_0.5_Al_2_O_4_/Kaol composite pigment with a bright blue color presents excellent thermal stability (Supplementary Figure S4; the Raman spectrum of Co_0.5_Ca_0.5_Al_2_O_4_/Kaol in ceramic glaze revealed that the composite pigments had a spinel structure) and a benign application prospect in coloring ceramics.

### 3.4 Formation mechanism

**SCHEME 1:**
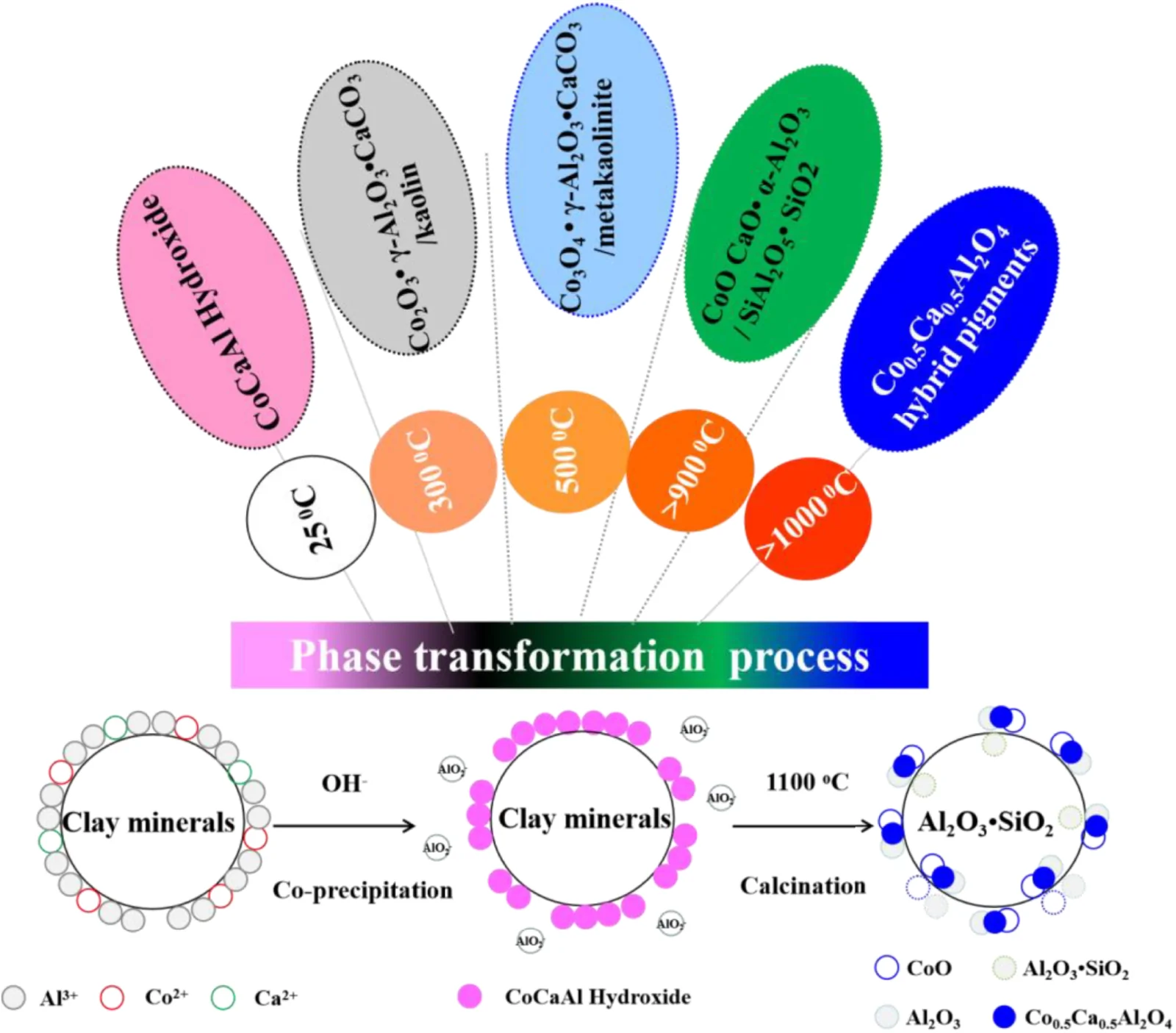
Schematic for the formation of the Co_0.5_Ca_0.5_Al_2_O_4_/Kaol composite pigment.

The possible formation mechanism of the Co_0.5_Ca_0.5_Al_2_O_4_/Kaol composite pigment was proposed according to the aforementioned analysis and is illustrated in Scheme 1. During the coprecipitation reaction, Co^2+^, Ca^2+^, and Al^3+^ were first adsorbed on the surface of Kaol due to the electrostatic interaction and ion exchange between the incorporated metal ions and Kaol. Then, Al^3+^ was transformed into Al(OH)_3_ at low pH and adsorbed on the surface of Kaol after the introduction of OH^−^. With the continuous increase in the pH, Co^2+^, Ca^2+^, and Al^3+^ were transformed into cobalt–calcium–aluminum hydroxides (Co–Ca–Al hydroxides), which was first transformed into γ-AlO(OH)·CaCO_3_·Co_3_O_4_/Kaol in the subsequent high-temperature crystallization process (Akdemir et al., 2011). As the temperature increased to approximately 500 °C, γ-AlO(OH) was converted into highly amorphous Al_2_O_3_. With an increase in the calcining temperature, Kaol was transformed into metakaolinite (2SiO_2_·Al_2_O_3_) at 700 °C and Co_3_O_4_ was progressively transformed into the CoAl_2_O_4_ phase above 800 °C, in which CaCO_3_ was thermally decomposed into CaO at 900 °C; the Co_3_O_4_ phase completely vanished as the calcining temperature was above 1,000 °C. Finally, the produced Ca, Co, and Al species reacted with each other to form the Co_0.5_Ca_0.5_Al_2_O_4_ composite pigment, and the metakaolinite was turned into mullite at 1,100 °C (Cava et al., 2006; Zhang et al., 2019). The thermal reduction of Co^3+^ to Co^2+^ and the diffusion and reorganization of Co^2+^, Ca^2+^, and Al^3+^ ions originating from the precursor and Kaol were simultaneously conducted.

## 4 Conclusion

By integration of metal ion doping and clay mineral loading, a series of Co_x_M_1−x_Al_2_O_4_/Kaol composite pigments with high blue intensity was successfully prepared via coprecipitation and a subsequent high-temperature crystallization process based on CoAl_2_O_4_. It was revealed that the incorporated cheap metal ions and Kaol obviously decreased cobalt consumption and enhanced the color properties of the cobalt blue pigments. Furthermore, the color of the composite pigments could be facilely adjusted by incorporating different metal elements. After the incorporation of Ca^2+^, the obtained composite pigments presented a blue color with a red hue, but Co_x_Zn_1−x_Al_2_O_4_ composite pigments were blue with a green hue. It is worth mentioning that the dispersion of pigment particles was significantly improved, and the color properties were superior to those of reported works and commercial cobalt blue pigments. Therefore, this study is expected to provide a feasible strategy for the preparation of low-cost and high-grade cobalt blue pigments compared with traditional solid- or liquid-phase preparation methods.

## Data Availability

The original contributions presented in the study are included in the article/Supplementary Material, further inquiries can be directed to the corresponding author.
