# Supplementary material for: Preparation of CoxM1−xAl2O4/kaolinite (M = Zn, Ca, and Mg) composite pigments for ceramic glaze
Source: Front Chem. 2026 Sep 1;14:1879833. doi: 10.3389/fchem.2026.1879833 (PMC13574595; doi:10.3389/fchem.2026.1879833)
Supplement: Supplementary file 1 [file DataSheet1.doc]

**Preparation of** **Co_x_M_1-x_Al_2_O_4_/****kaolinite (M = Zn, Ca, Mg) composite pigments for ceramic glaze**

Anjie Zhang^a,b,c*^, Mengmeng Wang^a^, Xiangwei Xiao^a^, Wenqiang Yan^a^, Hao Yang^d^, Bin Mu^e^, Aiqin Wang^e^

*^a^ College of Civil Engineering, Northwest Minzu University, Lanzhou 730000, P. R. China*

*^b^ Key Laboratory of Green Engineering Materials and Low-carbon Construction，Gansu Province*

*^c^ Industrial Research Institute of Prefabricated Buildings and Energy-Saving Materials*

*^d^ School of Chemical Engineering, Zhengzhou University, Zhengzhou 450001, China*

*^e^ Key Laboratory of Clay Minerals of Gansu Province, Research Center of Resource Chemistry and Energy Materials, Lanzhou Institute of Chemical Physics, Chinese Academy of Sciences, Lanzhou 730000, P. R. China*

**Table S1 Chemical compositions of Kaol before and after being treated using HCl**

| Kaol | Al_2_O_3_(%) | Na_2_O(%) | MgO(%) | CaO(%) | SiO_2_(%) | K_2_O(%) | Fe_2_O_3_(%) |
| --- | --- | --- | --- | --- | --- | --- | --- |
| Before | 27.45 | 0.17 | 0.52 | 0.07 | 43.12 | 1.3 | 0.18 |
| After | 27.43 | 0.23 | 0.38 | 0.01 | 48.71 | 3.55 | 0.37 |

**Table S2** The XRF of the Co_0.5_Ca_0.5_Al_2_O_4_/Kaol composite pigment

| Sample | Element (Wt %) | | | | | | | | |
| --- | --- | --- | --- | --- | --- | --- | --- | --- | --- |
|  | O | Al | Si | Co | Ca | Mg | K | Na | total |
| Co_0.5_Ca_0.5_Al_2_O_4_/Kaol | 36.27 | 25.41 | 13.47 | 11.87 | 9.62 | 1.24 | 0.89 | 1.23 | 100 |
| CoAl_2_O_4_/Kaol | 38.51 | 26.95 | 8.59 | 22.13 | 0.05 | 1.31 | 1.07 | 1.39 | 100 |
| CoAl_2_O_4_ | 38.09 | 29.34 | - | 32.57 | - | - | - | - | 100 |

**Table S3** Comparison of the color parameters of CoAl_2_O_4_ pigments prepared in this study with the reported and commercial ones.

| Preparation method | Temperature | *L^*^* | *a^*^* | *b^*^* | References |
| --- | --- | --- | --- | --- | --- |
| Ultrasonic hydrothermal method | 250°C | 42.3 | -5.8 | -26.1 | (Sale 2015) |
| Co-precipitation method | 1000°C | 53.43 | 4.75 | 41.78 | (Zheng et al., 2015) |
| Sol-Gel | 1100°C | 43.79 | -18.24 | -22.25 | (Peymannia et al., 2015) |
| Pechini-sacrificial agent method | 950°C | 41.76 | -6.57 | -45.01 | (Wang et al., 2016) |
| Polymeric precursor method | 900°C | 47.47 | -3.19 | -36.24 | (De Souza et al., 2009) |
| Co-precipitation method | 1200°C | 44.3 | -13.6 | -42.9 | (Melo et al., 2003) |
| Co-precipitation method | 1100°C | 54.6 | -2.5 | -50.4 | (Zhang et al., 2017a) |
| Co-precipitation method | 1100°C | 34.32 | 10.39 | -65.84 | This work |
| Commercial product-1 | - | 37.12 | -0.4 | -59.37 | - |
| Commercial product-2 | - | 33.91 | 1.66 | -58.1 | - |

**Table S4 the CIE of Co_1-x_Ca_x_Al_2_O_4_ at different calcining temperature**

| Sample | Temperature (^o^C) | L^*^ | a^*^ | b^*^ | C^*^ | H^*^ |
| --- | --- | --- | --- | --- | --- | --- |
| Co_0.1_Ca_0.9_Al_2_O_4_ | 800 | 63.61 | -6.11 | -24.50 | 25.28 | 256.01 |
| Co_0.2_Ca_0.8_Al_2_O_4_ | 800 | 63.61 | -6.11 | -24.52 | 29.20 | 252.21 |
| Co_0.3_Ca_0.7_Al_2_O_4_ | 800 | 49.89 | -8.92 | -27.80 | 35.16 | 249.79 |
| Co_0.4_Ca_0.6_Al_2_O_4_ | 800 | 36.76 | -12.14 | -32.99 | 35.45 | 241.84 |
| Co_0.5_Ca_0.5_Al_2_O_4_ | 800 | 29.04 | -16.73 | -31.25 | 36.00 | 236.35 |
| Co_0.6_Ca_0.4_Al_2_O_4_ | 800 | 24.60 | -18.83 | -28.30 | 30.69 | 238.52 |
| Co_0.7_Ca_0.3_Al_2_O_4_ | 800 | 16.43 | -16.02 | -26.16 | 26.87 | 226.00 |
| Co_0.8_Ca_0.2_Al_2_O_4_ | 800 | 12.01 | -18.66 | -19.32 | 24.25 | 232.28 |
| Co_0.9_Ca_0.1_Al_2_O_4_ | 800 | 9.15 | -14.83 | -19.18 | 21.00 | 216.64 |
| CoAl_2_O_4_ | 800 | 7.51 | -16.85 | -12.53 | 27.40 | 213.12 |
| Co_0.1_Ca_0.9_Al_2_O_4_ | 900 | 63.53 | -5.72 | -32.32 | 32.82 | 259.97 |
| Co_0.2_Ca_0.8_Al_2_O_4_ | 900 | 49.36 | -5.77 | -43.23 | 43.62 | 262.40 |
| Co_0.3_Ca_0.7_Al_2_O_4_ | 900 | 39.58 | -6.16 | -48.34 | 48.73 | 262.73 |
| Co_0.4_Ca_0.6_Al_2_O_4_ | 900 | 34.43 | -8.00 | -48.41 | 49.07 | 260.61 |
| Co_0.5_Ca_0.5_Al_2_O_4_ | 900 | 28.87 | -5.72 | -47.04 | 50.38 | 263.07 |
| Co_0.6_Ca_0.4_Al_2_O_4_ | 900 | 25.8 | -6.17 | -44.17 | 44.6 | 262.05 |
| Co_0.7_Ca_0.3_Al_2_O_4_ | 900 | 24.65 | -11.15 | -38.87 | 40.44 | 254.00 |
| Co_0.8_Ca_0.2_Al_2_O_4_ | 900 | 25.7 | -11.90 | -39.92 | 41.66 | 253.40 |
| Co_0.9_Ca_0.1_Al_2_O_4_ | 900 | 20.69 | -13.93 | -33.42 | 36.21 | 247.37 |
| CoAl_2_O_4_ | 900 | 23.56 | -19.32 | -32.11 | 37.48 | 238.97 |
| Co_0.1_Ca_0.9_Al_2_O_4_ | 1000 | 63.17 | -4.52 | -39.65 | 39.91 | 263.49 |
| Co_0.2_Ca_0.8_Al_2_O_4_ | 1000 | 51.95 | -1.04 | -52.20 | 52.21 | 268.86 |
| Co_0.3_Ca_0.7_Al_2_O_4_ | 1000 | 39.95 | 4.21 | -63.59 | 63.73 | 273.79 |
| Co_0.4_Ca_0.6_Al_2_O_4_ | 1000 | 38.54 | 1.20 | -60.89 | 60.9 | 271.13 |
| Co_0.5_Ca_0.5_Al_2_O_4_ | 1000 | 33.85 | 4.41 | -59.96 | 61.12 | 274.20 |
| Co_0.6_Ca_0.4_Al_2_O_4_ | 1000 | 33.51 | 3.18 | -59.16 | 59.24 | 273.08 |
| Co_0.7_Ca_0.3_Al_2_O_4_ | 1000 | 29.29 | 2.76 | -54.73 | 54.80 | 272.89 |
| Co_0.8_Ca_0.2_Al_2_O_4_ | 1000 | 31.00 | 0.36 | -54.89 | 54.89 | 270.38 |
| Co_0.9_Ca_0.1_Al_2_O_4_ | 1000 | 27.48 | -3.82 | -48.44 | 48.59 | 265.49 |
| CoAl_2_O_4_ | 1000 | 26.94 | -7.43 | -45.37 | 45.97 | 260.7 |
| Co_0.1_Ca_0.9_Al_2_O_4_ | 1100 | 61.98 | -2.03 | -45.43 | 45.47 | 267.44 |
| Co_0.2_Ca_0.8_Al_2_O_4_ | 1100 | 50.58 | 0.70 | -56.33 | 56.34 | 270.71 |
| Co_0.3_Ca_0.7_Al_2_O_4_ | 1100 | 39.71 | 5.99 | -64.88 | 65.15 | 275.28 |
| Co_0.4_Ca_0.6_Al_2_O_4_ | 1100 | 36.94 | 8.46 | -65.52 | 66.06 | 277.36 |
| Co_0.5_Ca_0.5_Al_2_O_4_ | 1100 | 34.32 | 10.39 | -65.84 | 66.67 | 279.10 |
| Co_0.6_Ca_0.4_Al_2_O_4_ | 1100 | 32.71 | 12.25 | -64.81 | 65.95 | 280.71 |
| Co_0.7_Ca_0.3_Al_2_O_4_ | 1100 | 30.70 | 12.29 | -62.89 | 64.08 | 281.05 |
| Co_0.8_Ca_0.2_Al_2_O_4_ | 1100 | 30.70 | 10.07 | -61.44 | 62.26 | 279.31 |
| Co_0.9_Ca_0.1_Al_2_O_4_ | 1100 | 27.81 | 9.32 | -58.04 | 58.79 | 279.13 |
| CoAl_2_O_4_ | 1100 | 28.21 | 3.10 | -54.13 | 54.21 | 273.27 |
| Co_0.1_Ca_0.9_Al_2_O_4_ | 1200 | 57.15 | -4.17 | -47.27 | 47.46 | 264.95 |
| Co_0.2_Ca_0.8_Al_2_O_4_ | 1200 | 47.01 | -2.58 | -55.35 | 55.41 | 267.33 |
| Co_0.3_Ca_0.7_Al_2_O_4_ | 1200 | 38.44 | 1.33 | -60.94 | 60.95 | 271.25 |
| Co_0.4_Ca_0.6_Al_2_O_4_ | 1200 | 32.39 | 10.20 | -62.70 | 63.52 | 279.25 |
| Co_0.5_Ca_0.5_Al_2_O_4_ | 1200 | 29.65 | 13.69 | -63.44 | 63.92 | 282.37 |
| Co_0.6_Ca_0.4_Al_2_O_4_ | 1200 | 28.99 | 15.31 | -62.89 | 64.73 | 283.69 |
| Co_0.7_Ca_0.3_Al_2_O_4_ | 1200 | 26.61 | 17.26 | -61.60 | 63.97 | 285.66 |
| Co_0.8_Ca_0.2_Al_2_O_4_ | 1200 | 27.79 | 14.08 | -60.82 | 62.43 | 283.04 |
| Co_0.9_Ca_0.1_Al_2_O_4_ | 1200 | 25.31 | 15.53 | -59.11 | 61.12 | 284.72 |
| CoAl_2_O_4_ | 1200 | 24.74 | 14.33 | -57.71 | 59.46 | 283.95 |

**Table S5 the CIE of Co_1-x_Zn_x_Al_2_O_4_ at different calcining temperature**

| Sample | Temperature (^o^C) | L^*^ | a^*^ | b^*^ | C^*^ | H^*^ |
| --- | --- | --- | --- | --- | --- | --- |
| Co_0.1_Zn_0.9_Al_2_O_4_ | 800 | 65.64 | -12.82 | -1.54 | 12.91 | 186.83 |
| Co_0.2_Zn_0.8_Al_2_O_4_ | 800 | 52.12 | -12.28 | 15.60 | 19.86 | 128.20 |
| Co_0.3_Zn_0.7_Al_2_O_4_ | 800 | 41.18 | -18.8 | 12.34 | 22.48 | 146.72 |
| Co_0.4_Zn_0.6_Al_2_O_4_ | 800 | 29.18 | -23.48 | 8.24 | 24.88 | 160.67 |
| Co_0.5_Zn_0.5_Al_2_O_4_ | 800 | 21.95 | -31.55 | 7.14 | 32.34 | 167.26 |
| Co_0.6_Zn_0.4_Al_2_O_4_ | 800 | 14.82 | -34.76 | 2.72 | 34.87 | 175.54 |
| Co_0.7_Zn_0.3_Al_2_O_4_ | 800 | 13.88 | -32.81 | -0.57 | 32.81 | 181.00 |
| Co_0.8_Zn_0.2_Al_2_O_4_ | 800 | 9.80 | -25.52 | -6.20 | 26.26 | 193.65 |
| Co_0.9_Zn_0.1_Al_2_O_4_ | 800 | 15.04 | -28.07 | -11.03 | 30.16 | 201.46 |
| CoAl_2_O_4_ | 800 | 7.513 | -16.85 | -12.53 | 27.40 | 213.12 |
| Co_0.1_Zn_0.9_Al_2_O_4_ | 900 | 60.09 | -12.92 | -24.6 | 27.79 | 242.29 |
| Co_0.2_Zn_0.8_Al_2_O_4_ | 900 | 50.19 | -18.03 | -3.39 | 18.34 | 190.64 |
| Co_0.3_Zn_0.7_Al_2_O_4_ | 900 | 44.32 | -20.23 | -23.06 | 30.68 | 228.73 |
| Co_0.4_Zn_0.6_Al_2_O_4_ | 900 | 37.75 | -21.77 | -29.43 | 36.61 | 233.52 |
| Co_0.5_Zn_0.5_Al_2_O_4_ | 900 | 30.13 | -23.47 | -31.45 | 39.24 | 233.27 |
| Co_0.6_Zn_0.4_Al_2_O_4_ | 900 | 30.58 | -23.44 | -31.23 | 39.05 | 233.1 |
| Co_0.7_Zn_0.3_Al_2_O_4_ | 900 | 28.71 | -22.83 | -32.59 | 39.79 | 234.99 |
| Co_0.8_Zn_0.2_Al_2_O_4_ | 900 | 24.62 | -20.04 | -32.32 | 38.03 | 238.2 |
| Co_0.9_Zn_0.1_Al_2_O_4_ | 900 | 26.62 | -22.03 | -32.13 | 38.96 | 235.57 |
| CoAl_2_O_4_ | 900 | 23.56 | -19.32 | -32.11 | 37.48 | 238.97 |
| Co_0.1_Zn_0.9_Al_2_O_4_ | 1000 | 59.88 | -9.09 | -37.44 | 38.53 | 256.36 |
| Co_0.2_Zn_0.8_Al_2_O_4_ | 1000 | 53.89 | -12.92 | -36.76 | 38.96 | 250.63 |
| Co_0.3_Zn_0.7_Al_2_O_4_ | 1000 | 43.15 | -12.50 | -45.90 | 47.57 | 254.77 |
| Co_0.4_Zn_0.6_Al_2_O_4_ | 1000 | 39.09 | -12.04 | -50.09 | 51.52 | 256.48 |
| Co_0.5_Zn_0.5_Al_2_O_4_ | 1000 | 38.31 | -12.01 | -50.18 | 51.60 | 256.54 |
| Co_0.6_Zn_0.4_Al_2_O_4_ | 1000 | 33.64 | -11.81 | -47.28 | 48.74 | 255.98 |
| Co_0.7_Zn_0.3_Al_2_O_4_ | 1000 | 32.92 | -8.98 | -49.70 | 50.50 | 259.76 |
| Co_0.8_Zn_0.2_Al_2_O_4_ | 1000 | 30.34 | -7.62 | -48.44 | 49.03 | 261.07 |
| Co_0.9_Zn_0.1_Al_2_O_4_ | 1000 | 32.02 | -8.78 | -49.11 | 49.89 | 259.86 |
| CoAl_2_O_4_ | 1000 | 26.94 | -7.43 | -45.37 | 45.97 | 260.7 |
| Co_0.1_Zn_0.9_Al_2_O_4_ | 1100 | 58.95 | -6.27 | -54.82 | 46.58 | 262.27 |
| Co_0.2_Zn_0.8_Al_2_O_4_ | 1100 | 54.10 | -8.48 | -58.84 | 48.42 | 259.91 |
| Co_0.3_Zn_0.7_Al_2_O_4_ | 1100 | 45.10 | -6.29 | -64.66 | 56.06 | 263.56 |
| Co_0.4_Zn_0.6_Al_2_O_4_ | 1100 | 39.49 | -1.93 | -65.41 | 59.19 | 268.14 |
| Co_0.5_Zn_0.5_Al_2_O_4_ | 1100 | 37.63 | -0.04 | -65.75 | 59.70 | 269.96 |
| Co_0.6_Zn_0.4_Al_2_O_4_ | 1100 | 35.21 | -0.44 | -62.29 | 57.54 | 269.56 |
| Co_0.7_Zn_0.3_Al_2_O_4_ | 1100 | 35.51 | 2.36 | -63.50 | 60.26 | 272.25 |
| Co_0.8_Zn_0.2_Al_2_O_4_ | 1100 | 31.60 | 4.15 | -61.09 | 58.07 | 274.10 |
| Co_0.9_Zn_0.1_Al_2_O_4_ | 1100 | 33.97 | 2.50 | -60.62 | 59.27 | 272.42 |
| CoAl_2_O_4_ | 1100 | 28.21 | 3.10 | -54.13 | 54.21 | 273.27 |
| Co_0.1_Zn_0.9_Al_2_O_4_ | 1200 | 54.1 | -2.62 | -46.16 | 54.88 | 267.26 |
| Co_0.2_Zn_0.8_Al_2_O_4_ | 1200 | 44.00 | -3.06 | -47.67 | 58.92 | 267.02 |
| Co_0.3_Zn_0.7_Al_2_O_4_ | 1200 | 40.94 | 4.32 | -55.70 | 64.80 | 273.82 |
| Co_0.4_Zn_0.6_Al_2_O_4_ | 1200 | 35.06 | 11.72 | -59.15 | 66.45 | 280.16 |
| Co_0.5_Zn_0.5_Al_2_O_4_ | 1200 | 32.10 | 14.65 | -59.7 | 66.39 | 282.75 |
| Co_0.6_Zn_0.4_Al_2_O_4_ | 1200 | 28.63 | 15.91 | -57.54 | 64.29 | 284.33 |
| Co_0.7_Zn_0.3_Al_2_O_4_ | 1200 | 31.45 | 13.14 | -60.21 | 64.85 | 281.69 |
| Co_0.8_Zn_0.2_Al_2_O_4_ | 1200 | 27.09 | 16.16 | -57.92 | 63.19 | 284.81 |
| Co_0.9_Zn_0.1_Al_2_O_4_ | 1200 | 30.06 | 10.50 | -59.21 | 61.52 | 279.82 |
| CoAl_2_O_4_ | 1200 | 24.74 | 14.33 | -57.71 | 59.46 | 283.95 |

**Table S6 the CIE of Co_1-x_Mg_x_Al_2_O_4_ at different calcining temperature**

| Sample | Temperature (^o^C) | L^*^ | a^*^ | b^*^ | C^*^ | H^*^ |
| --- | --- | --- | --- | --- | --- | --- |
| Co_0.1_Mg_0.9_Al_2_O_4_ | 800 | 66.49 | -7.77 | -12.73 | 14.91 | 238.62 |
| Co_0.2_Mg_0.8_Al_2_O_4_ | 800 | 48.78 | -15.86 | -19.09 | 24.82 | 230.28 |
| Co_0.3_Mg_0.7_Al_2_O_4_ | 800 | 38.47 | -20.53 | -15.95 | 26.00 | 217.86 |
| Co_0.4_Mg_0.6_Al_2_O_4_ | 800 | 28.14 | -25.87 | -13.99 | 29.41 | 208.4 |
| Co_0.5_Mg_0.5_Al_2_O_4_ | 800 | 23.66 | -28.54 | -15.21 | 32.34 | 208.05 |
| Co_0.6_Mg_0.4_Al_2_O_4_ | 800 | 22.64 | -30.51 | -8.54 | 31.68 | 195.63 |
| Co_0.7_Mg_0.3_Al_2_O_4_ | 800 | 9.40 | -27.02 | 1.02 | 27.03 | 177.83 |
| Co_0.8_Mg_0.2_Al_2_O_4_ | 800 | 13.49 | -29.46 | -6.89 | 30.25 | 193.16 |
| Co_0.9_Mg_0.1_Al_2_O_4_ | 800 | 11.73 | -26.51 | -9.92 | 28.30 | 200.51 |
| CoAl_2_O_4_ | 800 | 7.513 | -16.852 | -12.534 | 27.40 | 213.12 |
| Co_0.1_Mg_0.9_Al_2_O_4_ | 900 | 65.56 | -10.09 | -22.43 | 24.59 | 245.79 |
| Co_0.2_Mg_0.8_Al_2_O_4_ | 900 | 52.40 | -11.37 | -35.77 | 37.54 | 252.37 |
| Co_0.3_Mg_0.7_Al_2_O_4_ | 900 | 41.34 | -14.38 | -38.56 | 41.15 | 249.55 |
| Co_0.4_Mg_0.6_Al_2_O_4_ | 900 | 34.13 | -17.68 | -39.08 | 42.89 | 245.65 |
| Co_0.5_Mg_0.5_Al_2_O_4_ | 900 | 31.01 | -17.2 | -39.13 | 42.74 | 246.27 |
| Co_0.6_Mg_0.4_Al_2_O_4_ | 900 | 32.93 | -21.53 | -36.49 | 42.37 | 239.46 |
| Co_0.7_Mg_0.3_Al_2_O_4_ | 900 | 23.30 | -25.30 | -25.05 | 35.60 | 224.71 |
| Co_0.8_Mg_0.2_Al_2_O_4_ | 900 | 26.84 | -21.01 | -32.83 | 38.98 | 237.38 |
| Co_0.9_Mg_0.1_Al_2_O_4_ | 900 | 25.05 | -19.42 | -32.96 | 38.25 | 239.49 |
| CoAl_2_O_4_ | 900 | 23.56 | -19.32 | -32.11 | 37.48 | 238.97 |
| Co_0.1_Mg_0.9_Al_2_O_4_ | 1000 | 64.50 | -9.62 | -30.29 | 31.78 | 252.38 |
| Co_0.2_Mg_0.8_Al_2_O_4_ | 1000 | 52.66 | -8.06 | -46.11 | 46.81 | 260.09 |
| Co_0.3_Mg_0.7_Al_2_O_4_ | 1000 | 42.81 | -8.55 | -49.96 | 50.69 | 260.29 |
| Co_0.4_Mg_0.6_Al_2_O_4_ | 1000 | 38.20 | -8.69 | -52.82 | 53.53 | 260.66 |
| Co_0.5_Mg_0.5_Al_2_O_4_ | 1000 | 37.11 | -8.07 | -53.00 | 53.61 | 261.35 |
| Co_0.6_Mg_0.4_Al_2_O_4_ | 1000 | 36.65 | -10.77 | -50.81 | 51.94 | 258.03 |
| Co_0.7_Mg_0.3_Al_2_O_4_ | 1000 | 28.96 | -16.92 | -39.93 | 43.37 | 247.04 |
| Co_0.8_Mg_0.2_Al_2_O_4_ | 1000 | 31.70 | -10.55 | -47.28 | 48.44 | 257.42 |
| Co_0.9_Mg_0.1_Al_2_O_4_ | 1000 | 31.83 | -10.25 | -47.96 | 49.04 | 257.93 |
| CoAl_2_O_4_ | 1000 | 26.94 | -7.43 | -45.37 | 45.97 | 260.7 |
| Co_0.1_Mg_0.9_Al_2_O_4_ | 1100 | 60.19 | -7.15 | -59.82 | 43.55 | 260.55 |
| Co_0.2_Mg_0.8_Al_2_O_4_ | 1100 | 48.73 | -2.80 | -65.99 | 57.59 | 267.21 |
| Co_0.3_Mg_0.7_Al_2_O_4_ | 1100 | 42.58 | -1.76 | -66.61 | 60.86 | 268.34 |
| Co_0.4_Mg_0.6_Al_2_O_4_ | 1100 | 38.41 | 2.43 | -66.07 | 62.83 | 272.22 |
| Co_0.5_Mg_0.5_Al_2_O_4_ | 1100 | 38.22 | 2.35 | -66.42 | 62.94 | 272.14 |
| Co_0.6_Mg_0.4_Al_2_O_4_ | 1100 | 36.19 | 2.44 | -64.61 | 61.46 | 272.27 |
| Co_0.7_Mg_0.3_Al_2_O_4_ | 1100 | 30.25 | -4.87 | -52.93 | 51.25 | 264.55 |
| Co_0.8_Mg_0.2_Al_2_O_4_ | 1100 | 32.68 | 2.14 | -61.48 | 58.02 | 272.11 |
| Co_0.9_Mg_0.1_Al_2_O_4_ | 1100 | 31.87 | 3.84 | -60.63 | 58.52 | 273.76 |
| CoAl_2_O_4_ | 1100 | 28.21 | 3.10 | -54.13 | 54.21 | 273.27 |
| Co_0.1_Mg_0.9_Al_2_O_4_ | 1200 | 43.56 | -1.94 | -42.96 | 59.85 | 268.15 |
| Co_0.2_Mg_0.8_Al_2_O_4_ | 1200 | 39.99 | 6.49 | -57.52 | 66.31 | 275.61 |
| Co_0.3_Mg_0.7_Al_2_O_4_ | 1200 | 33.64 | 14.12 | -60.83 | 68.09 | 281.97 |
| Co_0.4_Mg_0.6_Al_2_O_4_ | 1200 | 32.48 | 14.86 | -62.78 | 67.72 | 282.68 |
| Co_0.5_Mg_0.5_Al_2_O_4_ | 1200 | 29.10 | 19.07 | -62.90 | 68.15 | 286.25 |
| Co_0.6_Mg_0.4_Al_2_O_4_ | 1200 | 31.77 | 13.47 | -61.41 | 66.00 | 281.78 |
| Co_0.7_Mg_0.3_Al_2_O_4_ | 1200 | 22.09 | 11.05 | -51.02 | 54.07 | 281.79 |
| Co_0.8_Mg_0.2_Al_2_O_4_ | 1200 | 29.30 | 12.59 | -57.98 | 62.76 | 281.58 |
| Co_0.9_Mg_0.1_Al_2_O_4_ | 1200 | 28.19 | 13.10 | -58.39 | 62.03 | 282.19 |
| CoAl_2_O_4_ | 1200 | 24.74 | 14.33 | -57.71 | 59.46 | 283.95 |

**Table S7** The performance parameter of the Co_0.5_Ca_0.5_Al_2_O_4_ composite pigment

| Item | results |
| --- | --- |
| Full Color/Diluted Color |  |
| Oil absorption，g/100g | 28-37 |
| Density，g/cm^3^ | 3.0-5.0 |
| PH value of the pigment aqueous suspension | 8-9 |
| Heat resistance， ^o^C | 1200 |
| Light resistance，Level 1-8 | 8 |
| Acid and alkali resistant，Level 1-5 | 5 |
| Weather resistance，Level 1-5 | 5 |
| Particle size，μm | 1-3 |

**Fig.S1** The XRD of Co_0.1_Ca_0.9_Al_2_O_4_/Kaol, Co_0.5_Ca_0.5_Al_2_O_4_/Kaol and JCPDs card of CoAl_2_O_4_, CaAl_2_O_4_, and mullite.

**Fig.S2** The reflectance spectra of Co_0.5_Ca_0.5_Al_2_O_4_/Kaol composite pigment at different temperatures and CoAl_2_O_4_ calcined at 1200 ^o^C

**Fig. S3** The CIE (a) and XRD (b) of Co_0.5_Ca_0.5_Al_2_O_4_/Kaol composite pigment at different calcined time

**Fig. S4** Raman spectrum of Co_0.5_Ca_0.5_Al_2_O_4_/Kaol in Ceramic glaze
